# 2D foam film coating of antimicrobial lysozyme amyloid fibrils onto cellulose nanopapers†

**DOI:** 10.1039/d3na00370a

**Published:** 2023-08-23

**Authors:** Nico Kummer, Luc Huguenin-Elie, Adrian Zeller, Yashoda Chandorkar, Jean Schoeller, Flavia Zuber, Qun Ren, Ashutosh Sinha, Kevin De France, Peter Fischer, Silvia Campioni, Gustav Nyström

**Affiliations:** a Laboratory for Cellulose & Wood Materials, Empa – Swiss Federal Laboratories for Materials Science and Technology Überlandstrasse 129, 8600 Dübendorf Switzerland gustav.nystroem@empa.ch silvia.campioni@empa.ch; b Institute of Food Nutrition and Health, ETH Zurich Schmelzbergstrasse 9 8092 Zurich Switzerland; c Laboratory for Biointerfaces, Empa – Swiss Federal Laboratories for Materials Science and Technology Lerchenfeldstrasse 5 9014 St. Gallen Switzerland; d Laboratory for Biomimetic Membranes and Textiles, Empa – Swiss Federal Laboratories for Materials Science and Technology Lerchenfeldstrasse 5 9014 St. Gallen Switzerland; e Institute for Biomechanics, ETH Zürich Stefano-Franscini-Platz 5 8093 Zürich Switzerland

## Abstract

Amyloid fibrils made from inexpensive hen egg white lysozyme (HEWL) are bio-based, bio-degradable and bio-compatible colloids with broad-spectrum antimicrobial activity, making them an attractive alternative to existing small-molecule antibiotics. Their surface activity leads to the formation of 2D foam films within a loop, similar to soap films when blowing bubbles. The stability of the foam was optimized by screening concentration and pH, which also revealed that the HEWL amyloid foams were actually stabilized by unconverted peptides unable to undergo amyloid self-assembly rather than the fibrils themselves. The 2D foam film was successfully deposited on different substrates to produce a homogenous coating layer with a thickness of roughly 30 nm. This was thick enough to shield the negative charge of dry cellulose nanopaper substrates, leading to a positively charged HEWL amyloid coating. The coating exhibited a broad-spectrum antimicrobial effect based on the interactions with the negatively charged cell walls and membranes of clinically relevant pathogens (*Staphylococcus aureus*, *Escherichia coli* and *Candida albicans*). The coating method presented here offers an alternative to existing techniques, such as dip and spray coating, in particular when optimized for continuous production. Based on the facile preparation and broad spectrum antimicrobial performance, we anticipate that these biohybrid materials could potentially be used in the biomedical sector as wound dressings.

## Abstract 2

The 2D lysozyme amyloid foam coating method can be used to deposit a 30 nanometer thick layer onto cellulose nanopapers introducing antimicrobial functionality.

## Graphical abstract

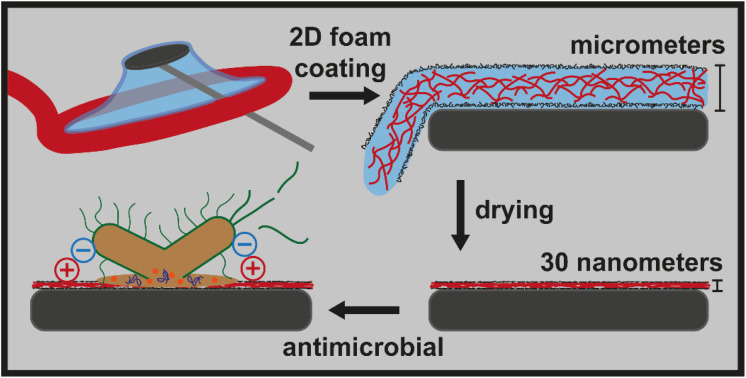

## Introduction

Due to its abundance, affordability, and antimicrobial activity hen egg white lysozyme (HEWL) is a promising candidate for the design of new materials to tackle the severe global health problem of antimicrobial resistance (AMR). Due to the excessive and incorrect use of small-molecule antibiotics, the evolutionary stress on bacteria has led to the emergence of resistant strains.^1,2^ Infections with multidrug-resistant strains are responsible for millions of deaths per year and pose a heavy financial burden on health care systems.^1,3^ As such, the development of new materials and implementation of advanced treatment strategies to combat AMR is critical.

Lysozyme naturally occurs in the body fluids of many organisms including humans and contributes to protection against pathogenic bacteria.^4,5^ This enzyme catalyzes the degradation of the outer cell wall of Gram-positive bacteria, thus mediating bacterial cell death. However, the antimicrobial potential of lysozyme is not limited to its enzymatic function.^6–8^ Due to its high isoelectric point, lysozyme (not only in the native state, but also in denatured states, which do not show any enzymatic activity) is positively charged at neutral/physiological pH, and can therefore interact with the negatively charged bacterial cell walls and membranes, leading to cellular aggregation and eventual cell lysis.^7–10^ Even aggregated forms of HEWL such as amyloid fibrils have demonstrated improved broad-spectrum antimicrobial activity, most likely due to a similar mechanism.^11–14^ Amyloid fibrils are a class of protein aggregates notorious for their role in neurodegenerative diseases such as Alzheimer's or Parkinson's.^15^ Research on disease-related misfolded proteins has revealed that they self-assemble into structurally similar morphologies, stabilized by a cross-β motif.^16^ This self-assembly of hydrogen-bonded antiparallel β-sheets provides excellent mechanical properties and environmental stability to amyloid fibrils. These properties are exploited by many organisms in nature, particularly microorganism such as bacteria (curli fibers) and fungi (hydrophobin rodlets), to fulfill some functions that are in this case beneficial.^17^ Even in humans, some examples of functional amyloids have been described, which are responsible for specific metabolic, storage and transport functions.^18^ In the past twenty years, the generic capability of polypeptide chains to self-assemble into amyloids under determined conditions has been used to target a wide range of applications such as water filtration, bioplastics, and biomedical engineering.^18–22^ In some cases, amyloid fibrils have been obtained from inexpensive food proteins under acidic conditions and at high temperature, leading to the hydrolysis of the full-length chain into short peptide fragments, some of which are capable to self-assemble into a cross-β-structure.^19^

So far, the antimicrobial potential of HEWL amyloid fibrils has been assessed in suspension and in the form of coatings on a variety of materials to create antimicrobial surfaces and wound dressings.^11,12,14,23–26^ HEWL amyloid fibrils show an enhanced antimicrobial effect and a broader spectrum of target organisms (Gram-positive and Gram-negative bacteria, fungi) compared to native HEWL (mostly active towards Gram-positive bacteria).^12,14^ This is attributed to both the accumulation of positive charge in the HEWL amyloid fibrils which enhances electrostatic interactions with the bacteria and the amphiphilic nature of amyloids which enables penetration into bacterial membranes.^11–14^ The antimicrobial properties of a material can be enhanced by coating its surface with the HEWL amyloid fibrils to expose as much positive charge as possible, rather than incorporating them into the bulk.^23^ Amyloid coatings (not only with HEWL amyloids) have been achieved using different methods such as vacuum filtration,^20,23^ dip coating,^12,27^ or Langmuir–Schaefer/Langmuir–Blodgett^22^ deposition.

In this work, we present a novel coating technique for amyloid fibrils, exploiting their tendency to adsorb at air–water interfaces (AWI) and lower the surface tension. Proteins are generally surface active due to their heterogeneous composition of amino acids, which vary in hydrophilicity.^28,29^ As such, amyloid fibrils have been shown to efficiently stabilize multiphase systems such as foams and emulsions.^30^ Foams are dispersions of air bubbles within a solvent phase, whereby the solvent tends to organize into lamellae, separating individual air bubbles. This foam formation phenomenon is commonly observed when blowing bubbles through a wand. In this situation, a single foam lamella can be generated by pulling a ring/loop out of a soap solution containing surface active molecules. Similarly, Zhang and co-workers developed a coating method using the surfactant sodium dodecyl sulphate (SDS) to stabilize such a 2D foam film inside a loop. By adding different nanomaterials (silica, polystyrene, titanium dioxide, gold nanoparticles, graphene oxide, ferritin, and nanocellulose) to the SDS solution and pulling a silicon (Si) wafer through the 2D foam film, the surface was efficiently and homogenously coated.^31^ In this work the HEWL amyloid fibrils serve as both the surface active compound stabilizing the AWI of the 2D foam film and the functional nanomaterial to be coated onto different substrates. The adsorption of amyloid fibrils at the AWI leads to dense networks and potentially introduces alignment into 2D nematic phases.^32^

Using mechanically strong nanopapers made from TEMPO-oxidized cellulose nanofibrils (TO-CNFs) as a substrate resulted in a transparent hybrid film composed of bio-based and bio-degradable materials.^33,34^ Nanocellulose is an ideal material for wound dressings, due to its high tensile strength, ability to absorb excessive liquid, while providing moisture to the wound and the possibility to incorporate drugs and nanomaterials.^35^ CNFs have been used and commercialized as wound dressing materials due to their excellent bio-compatibility and ability to promote wound healing.^36^ Combining bio-compatible^37^ HEWL amyloid fibrils with nanocellulose introduces additional antimicrobial functionality to these hybrid materials intended for wound dressing applications.^23,24^

Herein, we present a coating method for HEWL amyloid fibrils based on their surface activity, enabling the formation of 2D films within a loop. The stabilization of the 2D interfaces was optimized according to bulk (3D) foam stability tests. Factors such as amyloid concentration, pH, and the composition of the amyloid suspension were assessed, in relation to foam stability. The thickness of each HEWL amyloid layer deposited onto silicon wafers was determined by atomic force microscopy and ellipsometry. The HEWL amyloid fibrils were also successfully coated onto TO-CNF nanopapers, as confirmed by SEM, AFM, FTIR and XPS spectroscopy, and surface *ζ* potential measurements. Finally, the antimicrobial activity of the amyloid-coated TO-CNF nanopapers was tested.

## Materials and methods

### Materials

Hen egg white lysozyme (HEWL), (3-aminopropyl)triethoxysilane (APTES), 4-(2-hydroxyethyl)-1-piperazineethanesulfonic acid (HEPES), tryptic soy broth (TSB), and plant count agar (PC-agar) were purchased from Sigma-Aldrich. Hydrochloric acid (HCl), sodium hydroxide (NaOH), and sodium chloride (NaCl) were obtained from VWR. Glucose was provided by Fluka. All chemicals were used as received without any further purification. The TO-CNF were prepared according to a procedure published previously by our group.^38^

### Methods

#### Preparation of HEWL amyloid fibrils

The native HEWL (20 mg mL^−1^) was aggregated using acid hydrolysis at pH 2 and 90 °C. The samples were incubated in a Thermomixer (Eppendorf) for 24 hours under constant shaking (400 rpm). To quench the aggregation, the test tubes containing the freshly formed amyloid fibrils were cooled in an ice bath. Quantification of the conversion was performed using the method described in a previous publication by our group.^14^ The concentration of the peptide fraction after ultrafiltration was measured with UV-vis spectroscopy using the molar extinction coefficient of 38 000 M^−1^ cm^−1^.

#### Preparation of TO-CNF nanopapers

10 mL of 1.4 wt% TO-CNF suspension was dried at room temperature in a polystyrene Petri dish (diameter 60 mm) in a dust-free environment. Due to the slow drying at low temperature, the nanopapers were flat and suitable for coating. For further experiments, the nanopaper was cut into smaller pieces using a razor blade. The nanopapers were mechanically characterized in tensile tests performed according to a previous publication.^39^ The water sorption was tested gravimetrically by comparing the weight of a 1 × 1 cm^2^ piece of nanopaper before and after immersion in MilliQ water for 48 h.

#### Foam film coating method

Inspired by the wands/loops used to blow bubbles, a loop with a diameter of 5 cm was 3D printed. With this loop, it was possible to pull out an amyloid film from a polystyrene Petri dish filled with a 1 mg mL^−1^ amyloid suspension at pH 6.

#### Foam stability experiments

The foam stability of the different amyloid fractions, *i.e.* the original mixed system containing amyloid fibrils and peptides and the separated pure fibrils and peptides obtained by ultrafiltration was tested. The foam stability of the different fractions were assessed at different concentrations and pH in a custom-made foaming setup. For the measurements, 2 mL of the amyloid suspension was foamed through the frit of a chromatography column with a nitrogen flow of 20 cm^3^ min^−1^ to reach a column height of 8–10 cm. After the height was reached the main valve of the column was closed and the decay of the foam was monitored over time using a camera. An exponential decay function of the form*h*_foam_(*t*) = *h*_foam_(0)e^−*λt*^was fitted to the data, with *h*_foam_ being the foam height, *t* the time and *λ* the decay constant. The determined decay constant was used to calculate the foam half-life:
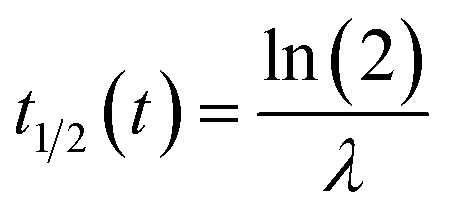


The half-lives were determined for triplicates of each sample and are shown as the mean half-life including the standard deviation.

#### Interfacial adsorption experiments

The adsorption of amyloids and peptides at the air–water interface (AWI) was measured with a Wilhelmy balance setup (KSV Nima) equipped with a Langmuir–Blodgett trough and movable barriers according to Bertsch and co-workers.^40^ After confirming the absence of contamination by closing the barriers without observing an increase in surface pressure, the trough was filled with 64.35 mL MilliQ water. The measurement was started after injecting 0.65 mL of amyloid mix and peptides adjusted to pH 6 and at a concentration of 1 mg mL^−1^. The final concentration of protein in the trough was 0.01 mg mL^−1^. The change in surface tension *γ*(*t*) compared to initial surface tension *γ*(0) is defined as the surface pressure *Π*(*t*) = *γ*(0) − *γ*(*t*) and was measured with a Wilhelmy plate made from filter paper.

#### Atomic force microscopy

All AFM images were obtained with a Bruker Icon 3 microscope equipped with RTESPA-300 probes for tapping mode. The resolution of all the images is 1024 × 1024 pixels, recorded at scan rates of 0.5 Hz. The thickness of the dry coating layers was measured on Si-wafers. After depositing 1, 2, and 3 layers of the foam film coating was scratched gently with a sterile syringe needle (without scratching the Si-wafer). The height difference between the Si-wafer and the coating was analyzed by extracting height profiles in 10 spots of three individual 10 × 10 μm^2^ scans. To account for the tilt in the images and to account for roughness, linear functions were fitted for the Si-wafer background and the coating layer. The height of the coating was calculated by subtracting the *y*-axis intercepts of the linear functions. AFM imaging of the coated and uncoated side of the TO-CNF nanopapers was performed on a dried sample that was immobilized on a glass slide using carbon tape.

#### Variable angle spectroscopic ellipsometry (VASE)

The thickness of the dry coating on SI wafers was also determined using ellipsometry to complement the AFM data with a non-invasive method. A JAW M2000 (J A Woollam) variable angle spectroscopic ellipsometer that was equipped with a He–Ne laser source (*λ* = 633 nm) was used for this purpose. The focusing lenses were at 50°, 60° and 70° angles from the surface normal and in a range of 370–1690 nm (0.7–3.3 eV) and an integration time of 5 s to record the amplitude (*ψ*) and the phase (Δ) components. Fitting of the raw data was performed using software CompleteEase based on a layered model using bulk dielectric functions for Si and SiO_2_ of the substrate Si wafer. The amyloid fibril layer was analyzed on the basis of a Cauchy model of the form *n* = *A* + *Bλ*^−2^, where *n* is the refractive index and *λ* is the wavelength, and *A* and *B* were considered to be 1.450 and 0.0103 for transparent organic polymeric films.

#### Scanning electron microscopy

The morphology of the TO-CNF nanopapers with and without the amyloid layer was investigated by SEM. The samples were coated with a layer of platinum (∼7 nm) before imaging with the FEI Quanta 650 FEG SEM (10.0 mm working distance, 5.0 kV accelerating voltage).

#### FTIR spectroscopy

The changes in the composition of the TO-CNF nanopapers with the amyloid coating was investigated *via* FTIR (Bruker Tensor 27 FT-IR) spectroscopy in attenuated total reflectance (ATR) mode. All spectra were recorded between 4000 and 600 cm^−1^ with a resolution of 4 cm^−1^ and 32 scans per sample. A background spectrum was measured before each measurement and subtracted from the sample spectra.

#### X-ray photoelectron spectroscopy

The atomical concentration at the surface of the specimens was measured using X-ray photoelectron spectroscopy (XPS). The measurements were performed on a Quantum 200 X-ray photoelectron spectrometer (Physical Electronics, MN, US) equipped with an Al Kα monochromatic source. Survey and high resolution spectra were acquired at a pass energy of 117.4 eV and 29.35 eV, respectively. Tougaard backgrounds were subtracted to measure peak intensities.

#### Suspension *ζ* potential measurements

The *ζ* potential measurements were conducted in triplicate utilizing a Malvern Zetasizer nano ZS with a folded capillary cell, and the reported values are the mean and standard deviation of the three measurements. The concentration of the TO-CNF and amyloid containing samples was 0.1 mg mL^−1^, the pure peptide sample (obtained by ultrafiltration, as described above) was measured at a concentration of 1 mg mL^−1^. All samples were measured in MilliQ water containing 1 mM NaCl for better conductivity during the measurements.

#### Surface *ζ* potential measurements

The surface *ζ* potential of the nanopapers was characterized using an Anton Paar Surpass 3 electrokinetic flow-through *ζ* potential instrument (Anton Paar, Austria). The dry film samples were slightly pre-wetted on the unexposed side of the nanopaper during the measurement and placed in a clamping cell. The system was rinsed with an electrolyte solution (1 mM KCl in milliQ at pH 7) and measured at the ideal gap height of 100–120 μm. The *ζ* potential was calculated using the formula
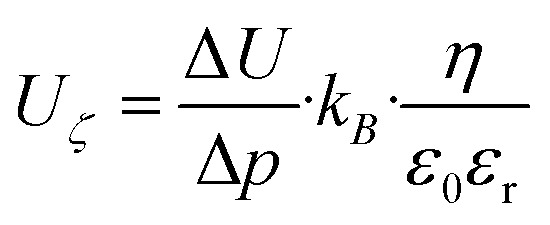
where Δ*U* is the potential difference, Δ*p* the gap-dependent pressure difference, *k*_B_ the Boltzmann constant, *η* the viscosity of the electrolyte, *ε*_0_ the permittivity in vacuum, and *ε*_r_ the relative permittivity of the electrolyte.

#### Antimicrobial activity testing

The coated TO-CNF nanopapers were tested for their antimicrobial activity against common pathogenic strains, namely Gram-positive *Staphylococcus aureus* (ATCC 6538), Gram-negative *Escherichia coli* (DSMZ 1576), and the fungus *Candida albicans* (ATCC 90028). The pre-cultures were prepared according to a previous publication,^14^ and diluted to optical densities of 0.01 (*S. aureus* and *E. coli*) and 0.1 (*C. albicans*) using 10 mM HEPES buffer at pH 7.2. The coated TO-CNF nanopapers were cut into samples with a diameter of 5 mm using a biopsy punching tool. The samples were placed into 48-well plates with the active amyloid coated side facing 5 μL of bacterial culture prepared at the bottom of each well. The samples were incubated for 4 h at room temperature in humidified air to prevent the drying of the samples. After the incubation, 200 μL HEPES buffer were added and the samples were sonicated for 5 min and vortexed for 15 s to detach the microorganisms. The 200 μL were then transferred to a fresh 96-well plate to prepare a dilution series down to 10^−3^. Aliquots of 20 μL were dropped onto PC-agar plates, and incubated at 37 °C overnight, before counting the colony forming units (CFU). Each sample was tested in triplicate, the antimicrobial activity is reported as the mean growth in CFU mL^−1^ including the standard deviation. The killing ratio was calculated as the ratio between the CFU of the coated sample and the uncoated TO-CNF controls.

## Results and discussion

### The 2D foam coating

**Fig. 1:**
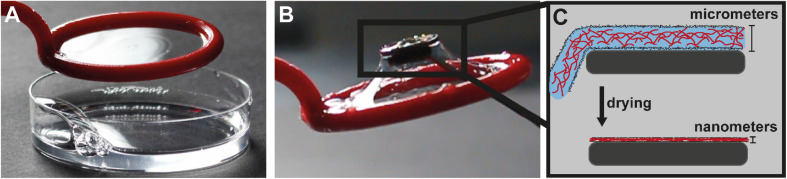
Photo of the 2D HEWL amyloid fibril film spanning the inside of a 3D printed loop (A). Transfer of the elastic 2D foam film onto a substrate (B). Schematic showing the micrometer thick wet deposited foam film compressing into a nanometer thick dried coating on a substrate (C).

When air is dispersed into a HEWL amyloid fibril suspension, for example by shaking, stable foams are formed. Occasionally, a big bubble spanning the entire rim of the vial can be observed after opening the lid. Seeing this bubble brought back childhood memories of blowing soap bubbles and gave inspiration to try to blow amyloid fibril bubbles. While creating a 2D amyloid-stabilized film was successful by pulling out the amyloid suspension using a custom-made loop (Fig. 1A), blowing the film into a bubble was unsuccessful, as the film burst upon blowing. However, the 2D film was successfully transferred onto a silicon wafer (Fig. 1B) and subsequently dried under ambient conditions to form a homogenous, ultra-thin HEWL amyloid fibril coating (Fig. 1C).

### Optimization of the foam stability

**Fig. 2:**
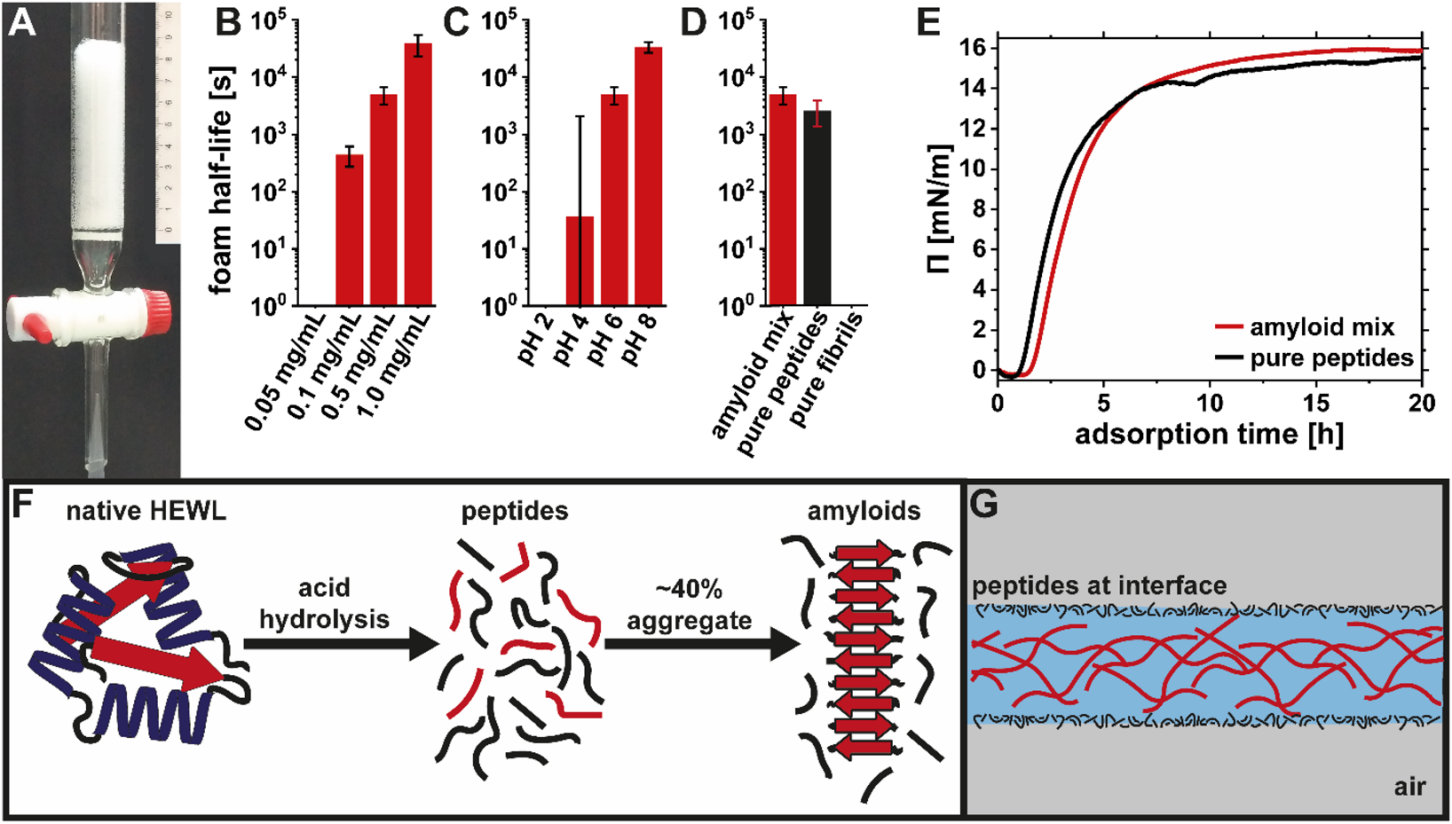
The setup used to measure foam stability consisted of a chromatography column. The samples were foamed by dispersing nitrogen through the frit (A). Amyloid mix foam half-life in dependence of concentration (B) and pH (C). The amyloid mix was separated into pure peptides and pure fibrils using ultrafiltration to measure their individual contribution to foam stability (D). Surface pressure measurement showing similar adsorption kinetics for the amyloid mix and the pure peptides (E). Amyloid self-assembly pathway using acid hydrolysis (F). Schematic illustrating that the air–water interface is stabilized by peptides, while the amyloid fibrils form a network within the foam lamella (G).

To optimize the stability of the 2D amyloid fibril foam film inside the loop, a foaming device was created using a fritted chromatography column (Fig. 2A). By streaming nitrogen at a fixed flux through the frit, the amyloid suspensions were foamed at different concentrations and pH values. At pH 6, the HEWL amyloid fibril foam stability was strongly dependent on the amyloid concentration with foam half-lives in the order of 1 min at 0.1 mg mL^−1^, 1 h at 0.5 mg mL^−1^, and several hours at 1 mg mL^−1^ (Fig. 2B). Fig. 2C shows the foam stability of the amyloid suspensions at 0.5 mg mL^−1^ and at different pH. At pH values lower or equal to 4, a foam was either not obtained at all or highly unstable. The foams only became stable at pH values of 6 and higher. However, at pH 8, small agglomerates of amyloid fibrils were visible to the naked eye (Fig. S1†). Finally, at constant pH of 6, different fractions, referred to as the original amyloid mix (consisting of 40% fibrils and 60% peptides^14^), and the pure fibrils and the pure peptides (separated by ultrafiltration), were compared (Fig. 2D). The foam half-lives of the amyloid mix and the pure peptides were similar, while the pure amyloid fibrils did not form stable foams. This finding was also confirmed with surface pressure (*Π*) measurements in Fig. 2E, which showed a similar adsorption isotherm for the amyloids mix and the peptide fraction alone. These two experiments lead to the conclusion that the peptides rather than the amyloid fibrils are the species responsible for interfacial stabilization. Fig. 2F illustrates the amyloid self-assembly pathway mediated by acid hydrolysis of the native protein into short peptide fragments. Only some of these take part in the self-assembly into amyloid fibrils, while the rest remain in the sample as free peptides. The conversion of HEWL into amyloid fibrils and possible purification strategies were discussed in detail in our previous publication.^14^ Based on the foam stability and surface pressure measurements (Fig. 2D and E), which are in good agreement with other findings from literature,^30^ we propose the stabilization of the air–water interface by the peptides (illustrated in Fig. 2G).

In agreement with results from amyloid systems made from soy protein isolate, it is thus not the actual amyloid fibrils that stabilize the AWI, but rather the free peptides.^30^ In addition to the much lower molecular weight of the peptides, which enables faster diffusion and adsorption to the interface compared to the fibrils,^30^ there might be two more factors promoting peptide adsorption. Peptides largely consist of a disordered/unfolded structure,^14^ with unfavorably exposed hydrophobic patches enhancing the amphiphilic properties and causing a higher surface activity. Moreover, it was shown that the unfolding of native HEWL adsorbed at the air–water interface (induced by the reduction of disulfide bond by the reducing agent dithiothreitol) resulted in more elastic interfacial layers.^41^ Additionally, the *ζ* potential of the peptides is lower than that of the fibrils (Fig. S2†), which reduces the adsorption barrier caused by the double layer repulsion between adsorbed peptides at the interface and peptides in the bulk. This adsorption barrier is also the reason for the reduced adsorption of the HEWL amyloid mix and the resultant weak foams at low pH. Below pH 4, the charge of the system is dominated by the repulsive forces between the different protonated basic amino acids (arginine, histidine and lysine). Negative charges are introduced with the deprotonation of the acidic amino acids, above their p*K*_a_ of around 4. Even though no drastic decrease in positive charge can be observed in the pH-dependent *ζ* potential measurements (Fig. S2†), the negative charge from the acidic amino acids will cause local attraction and facilitate peptide adsorption. At pH 6, the acidic amino acids are fully deprotonated and therefore the double layer repulsion and the connected adsorption barrier are reduced, enabling more stable foams. The half-life of the foam at pH 8 was even higher. However, aggregates in the 2D foam film could be seen by eye due to too much attraction, which would also cause inhomogeneity in the foam film coating. Therefore, the optimized conditions for the coating were an amyloid concentration of 1 mg mL^−1^ and pH 6. Additional treatment of the amyloid mix, such as dialysis or ultrafiltration to reduce the relative amount of peptides in the system were omitted, to have the full surface activity provided by the peptides. Note that the results below were all generated with the crude amyloid system (without further purification) and referred to as amyloids or amyloid fibrils.

### Thickness measurements on HEWL amyloid coating on silicon wafers

**Fig. 3:**
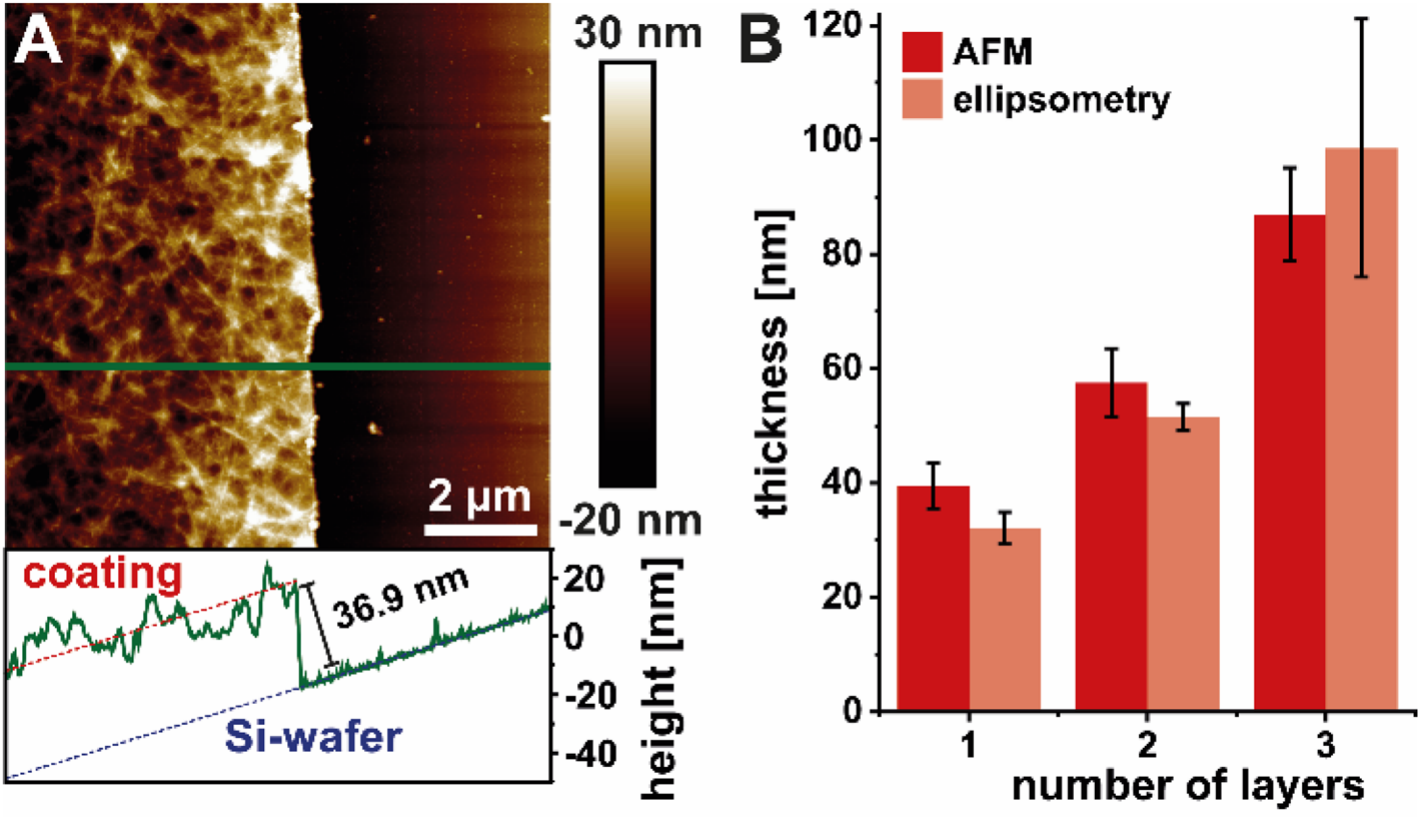
Thickness measurement of one amyloid fibril coating layer on Si-wafer by scratching off the coating on the right side of the image and measuring the step height using AFM (A). Comparison of thickness measurements using AFM and variable angle spectroscopic ellipsometry (VASE) (B).

Using the optimized conditions of the HEWL amyloid suspension, the stable 2D foam films were reproducibly coated onto Si-wafers and dried under ambient conditions. After the coating was completely dried, additional layers could be added to tune the thickness of the coating. The thickness of the coating after applying one, two, and three layers of HEWL amyloids was measured by AFM and ellipsometry (Fig. 3). The AFM image in Fig. 3A shows the coating on the left side, while the Si-wafer background exposed by scratching the coating with a syringe needle can be seen on the right side. The green line indicates the position at which the profile shown below the AFM image was measured. The coating thickness was measured by subtracting the average height of the background from the average height of the coating (including tilt correction). The measured thickness values as a function of number of coating layers determined by AFM and ellipsometry are summarized in Fig. 3B. This demonstrates that layer-by-layer deposition with drying steps between the individual coating steps resulted in a discrete increase in layer thickness of around 30 nm with each additional layer. Notably, drying artifacts such as the coffee ring effect are reduced due to the amyloid fibril network present in the deposited wet films (Fig. S3†). The fibril network maintains its structure and densifies as the apparent amyloid fibril concentration increases upon evaporation of water.

### Characterization of the HEWL amyloid fibril coating on TO-CNF nanopapers

**Fig. 4:**
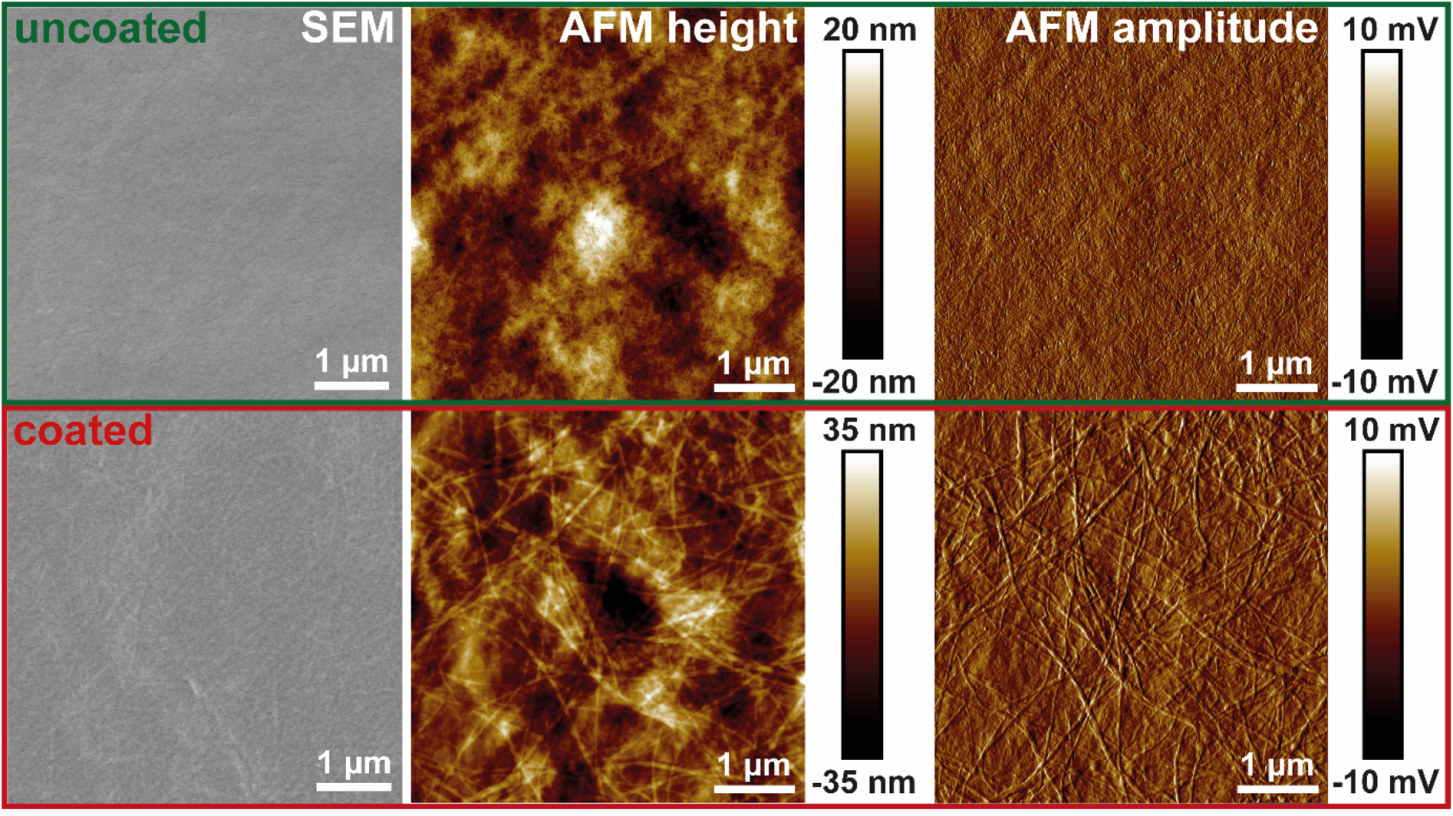
Morphology (SEM, AFM height and amplitude imaging) of TO-CNF nanopapers uncoated (upper panels) and coated (lower panels) with amyloid fibrils using the foam film coating method.

To create a fully bio-based and bio-degradable material with antimicrobial properties, the foam film coating was successfully applied to dried TO-CNF nanopapers. The stress–strain curves from tensile testing of the nanopaper (Fig. S4†) and a summary of the derived mechanical properties (Table S1†) are in good agreement with existing literature for mechanically strong dry nanocellulose films.^33,39^ The homogenous deposition of the foam film was confirmed with SEM and AFM imaging (Fig. 4). The smooth surface of the TO-CNF nanopaper was clearly covered with amyloid fibrils after drying the deposited amyloid layer.

**Fig. 5:**
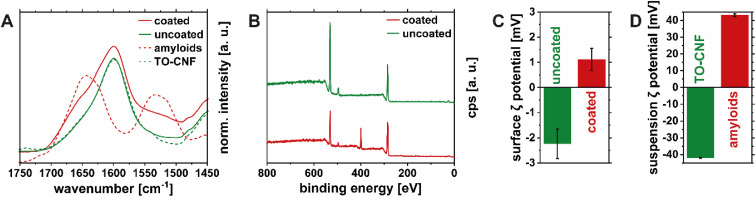
Surface characterization of TO-CNF nanopapers without (green) and with (red) the amyloid fibrils deposited with the foam film coating method. FTIR spectra of each side of the coated nanopaper (continuous lines) and reference spectra corresponding to pure TO-CNF and amyloids (dashed lines) (A). XPS spectra of uncoated and coated samples, with a nitrogen peak accounting for 13% of the elemental composition (B). Surface *ζ* potentials of uncoated and coated TO-CNF nanopapers (C) compared to *ζ* potentials of TO-CNF and amyloid suspensions at pH 7 (D).

Thickness measurements of the coating could not be performed on the TO-CNF substrate, as scratching only the amyloid layer was not feasible and because the refractive indices of protein and cellulose are too similar to perform meaningful ellipsometry measurements. Instead, the surface chemistry of the coated nanopapers was characterized using FTIR spectroscopy, XPS, and surface *ζ* potential measurements. Fig. 5A shows the IR spectrum of an uncoated TO-CNF nanopaper and after the deposition of the HEWL amyloid fibril coating in the region between 1450 and 1750 cm^−1^ (full spectra can be found in Fig. S5A and S5B†). The relevant peaks in this region are the C=O stretching vibration from deprotonated carboxyl groups of TO-CNF (1612 cm^−1^)^42^ and the amide I (1650 cm^−1^) and amide II (1550 cm^−1^) contributions of the HEWL amyloid coating.^43^ With the addition of the HEWL amyloid fibrils, a shoulder in the amide I region around 1650 cm^−1^ was observed, which can be attributed to the increase in protein content.^14,43^ The uncoated side of the TO-CNF nanopaper shows a slight increase of absorbance in the amide II region, which might be due to the absorption of excess liquid containing peptides into the bulk of the TO-CNF nanopaper. Due to the absorption of peptides into the sample and the penetration depth of the infrared radiation in the order of micrometers,^44^ protein was even detectable when measuring the uncoated side of the TO-CNF nanopaper. Fig. 5B shows XPS spectra providing complementary information about the chemistry at the immediate surface (penetration depth ∼ 10 nm).^45^ As expected, the spectrum for the uncoated side TO-CNF side of the nanopaper was dominated by the C 1s (284 eV), and O 1s (532 eV) peaks, whereas the N 1s (398 eV) appeared after depositing the HEWL amyloid fibril coating. The elemental composition of the coating layer was 69% carbon, 18% oxygen and 13% nitrogen, which are values typically found for protein layers.^46^ The uncoated side contained around 1% of nitrogen as well, which is likely due to the absorption of peptides mentioned above. Changes in the surface charge of the coated nanopapers were confirmed with surface *ζ* potential measurements (Fig. 5C). The negative potential (−2 mV) of the uncoated TO-CNF nanopapers turned positive after the deposition of the amyloid coating layer (+1 mV). Importantly, the *ζ* potential values from surface measurements and suspension measurements at pH 7 (Fig. 5D) are only comparable qualitatively and not in terms of absolute values. This is due to the reduced specific area of the measured surfaces as well as the different morphologies compared to the suspension and the fundamentally different measurement setup. Furthermore, the high pressure during the rinsing step and the surface *ζ* potential measurement might shear off some of the adsorbed amyloids on the surface of the TO-CNF resulting in underestimation of the surface *ζ*-potential.

The stability of the HEWL amyloid coating on the TO-CNF nanopapers was assessed by immersing a triplicate of samples in MilliQ water for 48 hours. Ultraviolet-visible (UV-vis) spectroscopy of the MilliQ water showed that the released material was mostly TO-CNF, which could also be seen by AFM imaging of the solution (Fig. S6†). Despite the lack of a pronounced protein signal in the UV-vis spectrum, some peptides could be seen in AFM as well, but no amyloid fibrils. These findings indicate that the TO-CNF nanopapers might not be stable enough for further use as wound dressing materials and require physical or chemical cross-linking to grant stability/integrity and improve the mechanical properties. However, AFM imaging of the amyloid-coated nanopapers after immersion in MilliQ water for 48 h confirmed the stability of the HEWL amyloid fibril coating (Fig. S7†). This can be explained by the good adhesion of the positively charged HEWL amyloids on the negatively charged TO-CNF,^47^ as well as the entanglement of the fibril network, allowing only for the release of unreacted peptides but not the amyloid fibrils.

The hybrid HEWL amyloid coated TO-CNF nanopaper could potentially be used as a wound dressing, due to its high tensile strength, biocompatibility, transparency allowing to monitor the wound and its ability to control the moisture of the wound.^36^ Another more cost-effective way to prepare a hybrid wound dressing using the HEWL-amyloid 2D foam coating could be its application onto other cellulose-based wound care materials like gauzes.^48^

### Antimicrobial activity of the HEWL amyloid fibril coating

**Fig. 6:**
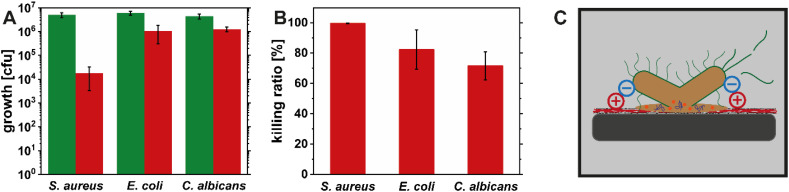
Antimicrobial activity of the uncoated TO-CNF nanopapers (green bars) and coated (red bars) with HEWL amyloid fibrils, expressed as microbial growth in CFU after 4 h of incubation (A). The same data expressed as killing ratio (B). Schematic illustrating the proposed mode of action based on electrostatic interactions between the positive charges of the coating and the negative cell walls and membranes of microorganisms leading to their rupture and the death of the microorganisms (C).

After confirming the successful and homogenous coating of TO-CNF nanopapers with the positively charged HEWL amyloid fibril layer, the antimicrobial activity of the hybrid material was investigated. The samples coated with HEWL amyloid fibrils were tested against *S. aureus* (Gram-positive bacteria), *E. coli* (Gram-negative bacteria) and *C. albicans* (pathogenic yeast). The TO-CNF nanopaper coated with HEWL amyloid fibrils showed a broad-spectrum antimicrobial effect against all the pathogens tested (Fig. 6A and B). The highest reduction of microbial growth, of almost 3 orders of magnitude compared to uncoated TO-CNF nanopaper (99.6% killing ratio), was observed against *S. aureus*. The reduction of *E. coli* and *C. albicans* was below 1 order of magnitude, corresponding to 82% and 72% killing efficiency, respectively. These results are qualitatively in good agreement with our previous study assessing antimicrobial potential of HEWL amyloid suspensions.^14^ A similar broad-spectrum antimicrobial activity was found in both cases, with the best activity against *S. aureus* and higher concentrations needed to inhibit the growth of *E. coli* and *C. albicans*, which is in agreement with other literature as well.^11,13^ A direct quantitative comparison of the amyloid suspensions and the coating surface is difficult, since the experimental approaches for testing the antimicrobial activity differ fundamentally. Due to the substantially reduced surface area in the coating layer compared to amyloids in suspension, the exposure of the bacteria to positively charged groups is decreased, which is also supported by the *ζ* potential data. However, the broad-spectrum antimicrobial effect can still be linked to the interactions between the positively charged amyloid fibrils and the negatively charged cell wall and cell membrane of the microbial cells (Fig. 6C). This antimicrobial mode of action based on positive charge has been proposed before for amyloid fibrils^11,13^ and seems to be a common feature among cationic polymers^49,50^ and also plays a role for the antimicrobial activity of cationic antimicrobial peptides.^51^ In our previous publications we observed antimicrobial activity of the peptide fraction.^14^ As discussed above, there is a release of peptides from the HEWL amyloid coating, which might also contribute to the antimicrobial effect through diffusion into the bulk medium.

## Conclusion

We report the successful use of 2D foam films based on HEWL amyloids as a coating method. The adsorption of HEWL amyloid fibrils at the air–water interface is concentration and pH dependent and actually dominated by the contribution of unconverted peptides present in the suspension. Therefore, the interfaces are effectively stabilized by the peptides, while the amyloid fibrils remain in the bulk of the foam lamella in the form of a network. The 2D foam film coating is a facile way of coating amyloid fibrils onto various substrates and offers tunability of the coating thickness by layer-by-layer deposition. The homogeneity of the coating was confirmed by SEM and AFM imaging, as well as spectroscopy techniques (FTIR and XPS). The 30 nm thick HEWL amyloid fibril coating is thick enough to shield the negative charge of the TO-CNFs as seen in surface *ζ* potential measurements of the coated nanopaper. Importantly, the HEWL amyloid coating resulted into functionalization of TO-CNF nanopaper, which showed broad-spectrum antimicrobial activity against *S. aureus* (Gram-positive), *E. coli* (Gram-negative) and *C. albicans* (pathogenic yeast) based on interactions between the positive charges of the HEWL amyloids and the negatively charged cell walls and membranes of the microbes leading to their disruption. The antimicrobial effect was strongest against *S. aureus* (3 log reduction of growth compared to uncoated TO-CNF nanopapers), while for *E. coli* and *C. albicans* the reduction was less than 1 log reduction. Our results indicate that for surface active proteins and protein aggregates the foam film coating potentially offers an alternative to other coating mechanisms, such as dip coating or spray coating, especially if it can be optimized to make the process continuous. The HEWL amyloid coated TO-CNF nanopapers are particularly attractive for their use as bio-based and, bio-compatible and bio-degradable wound dressing material, with an additional antimicrobial functionality. Overall, our findings offer a promising alternative to traditional coating methods and contribute to the development of innovative biomedical materials.

## Author contributions

Nico Kummer: conceptualization; data curation; formal analysis; investigation; methodology; project administration; validation; visualization; writing – original draft. Luc Huguenin-Elie: formal analysis; investigation; methodology; validation; writing – review & editing. Adrian Zeller: formal analysis; investigation; methodology; validation; writing – review & editing. Yashoda Chandorkar: formal analysis; investigation; methodology; validation; writing – review & editing. Jean Schoeller: formal analysis; investigation; methodology; validation; writing – review & editing. Flavia Zuber: formal analysis; investigation; methodology; validation; writing – review & editing. Qun Ren: methodology; supervision; validation; writing – review & editing. Ashutosh Sinha: formal analysis; investigation; methodology; validation; writing – review & editing. Kevin De France: formal analysis; investigation; methodology; validation; writing – review & editing. Peter Fischer: conceptualization; funding acquisition; methodology; supervision; writing – review & editing. Silvia Campioni: conceptualization; funding acquisition; project administration; supervision; writing – review & editing. Gustav Nyström: conceptualization; funding acquisition; project administration; supervision; writing – review & editing.

## Conflicts of interest

There are no conflicts to declare.

## Supplementary Material

NA-005-D3NA00370A-s001
