# Supplementary material for: 2D foam film coating of antimicrobial lysozyme amyloid fibrils onto cellulose nanopapers
Source: Nanoscale Adv. 2023 Aug 23;5(19):5276–85. doi: 10.1039/d3na00370a (PMC10521212; doi:10.1039/d3na00370a)
Supplement: NA-005-D3NA00370A-s001 [file NA-005-D3NA00370A-s001.pdf]

Electronic Supplementary Material (ESI) for Nanoscale Advances.
This journal is © The Royal Society of Chemistry 2023

Supplementary information for:

2D foam film coating of antimicrobial lysozyme amyloid fibrils onto cellulose nanopapers

Nico Kummer[a,b], Luc Huguenin-Elie[a], Adrian Zeller[a], Yashoda Chandorkar[c], Jean Schoeller[d,e], Flavia Zuber[c], Qun Ren[c], Ashutosh Sinha[a,b], Kevin De France[a,#], Peter Fischer[b], Silvia Campioni[a], Gustav Nyström*[a,b]*

[a]Laboratory for Cellulose & Wood Materials, Empa – Swiss Federal Laboratories for Materials Science and Technology, Überlandstrasse 129, 8600 Dübendorf, Switzerland

[b]Institute of Food Nutrition and Health, Schmelzbergstrasse 9, ETH Zurich, 8092 Zurich, Switzerland

[c]Laboratory for Biointerfaces, Empa – Swiss Federal Laboratories for Materials Science and Technology, Lerchenfeldstrasse 5, 9014 St. Gallen

[d]Laboratory for Biomimetic Membranes and Textiles, Empa – Swiss Federal Laboratories for Materials Science and Technology, Lerchenfeldstrasse 5, 9014 St. Gallen

[e]Institute for Biomechanics, Stefano-Franscini-Platz 5, ETH Zürich, 8093 Zürich, Switzerland

* To whom correspondence should be addressed

[#] present address: Department of Chemical Engineering, Queen's University, Kingston, Ontario K7L 3N6, Canada

E-Mail Gustav Nyström.: gustav.nystroem@empa.ch

E-Mail Silvia Campioni.: silvia.campioni@empa.ch

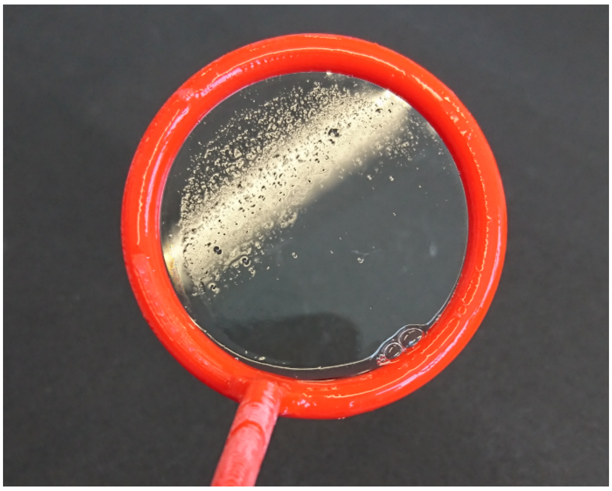

Figure S1: Photography of the loop with a HEWL amyloid 2D foam film at pH 8, showing inhomogeneity due to aggregation.

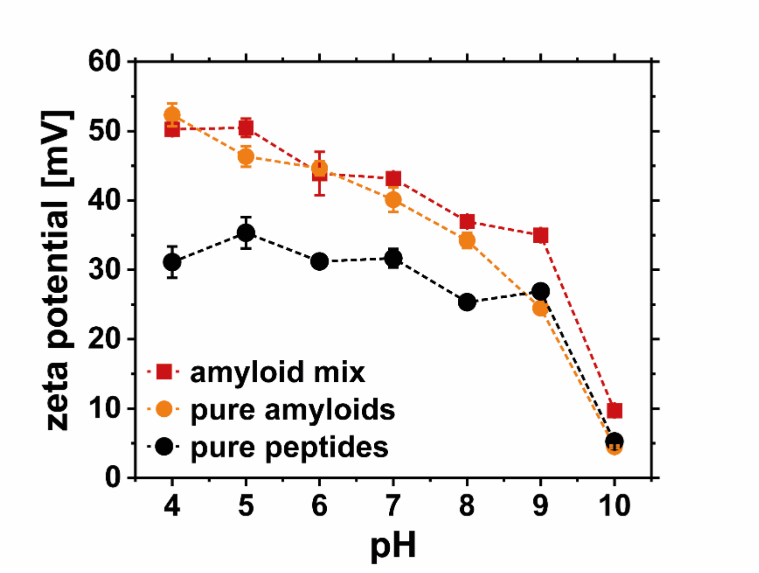

Figure S2: pH-dependent ζ potential of the amyloid mix and the pure amyloid fibrils and peptides separated by ultrafiltration.

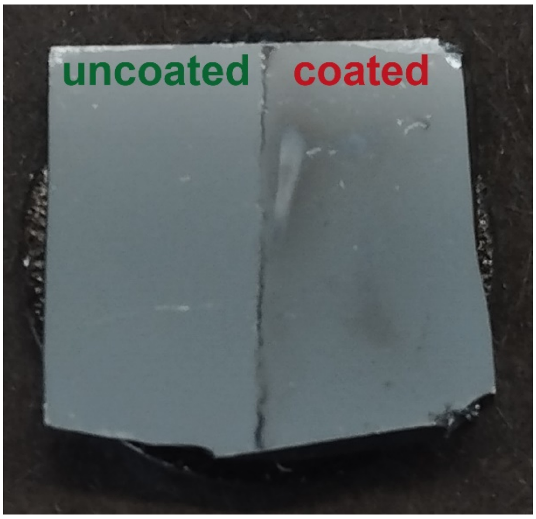

Figure S3: Photo of a silicon wafer that was covered with tape on the left side (uncoated) before depositing a layer of the 2D amyloid foam coating. After drying the tape was removed and the homogenous coating was visible on the right side (coated).

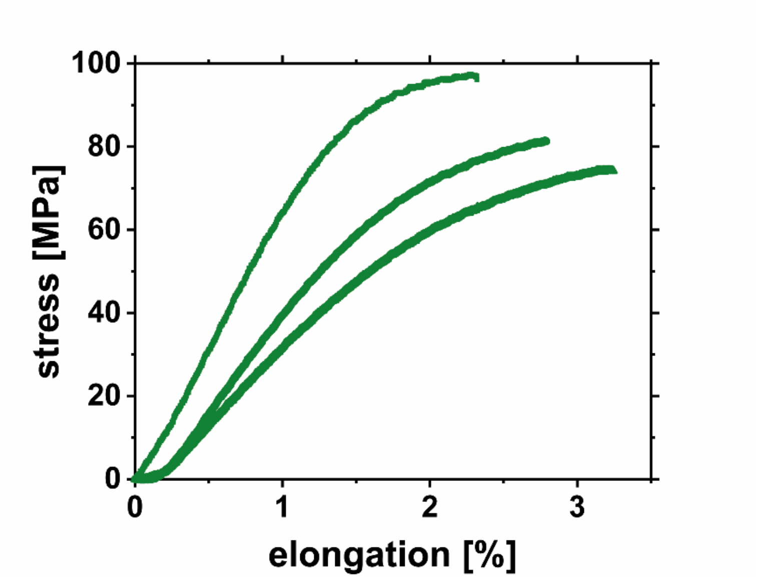

Figure S4: Stress-strain curves of the tensile tests performed on a triplicate of TO-CNF nanopaper.

Table S1: Summary of the result of the tensile tests including Elongation at break, modulus, tensile strength, and toughness, as well as water sorption behavior.

Elongation at Break	2.27 ± 0.24 %
Modulus	6.66 ± 2.21 GPa
Tensile Strength	84.40 ± 11.80 MPa
Toughness	1.38 ± 0.06 MJm^{-3}
Swelling	2320 ± 260 %

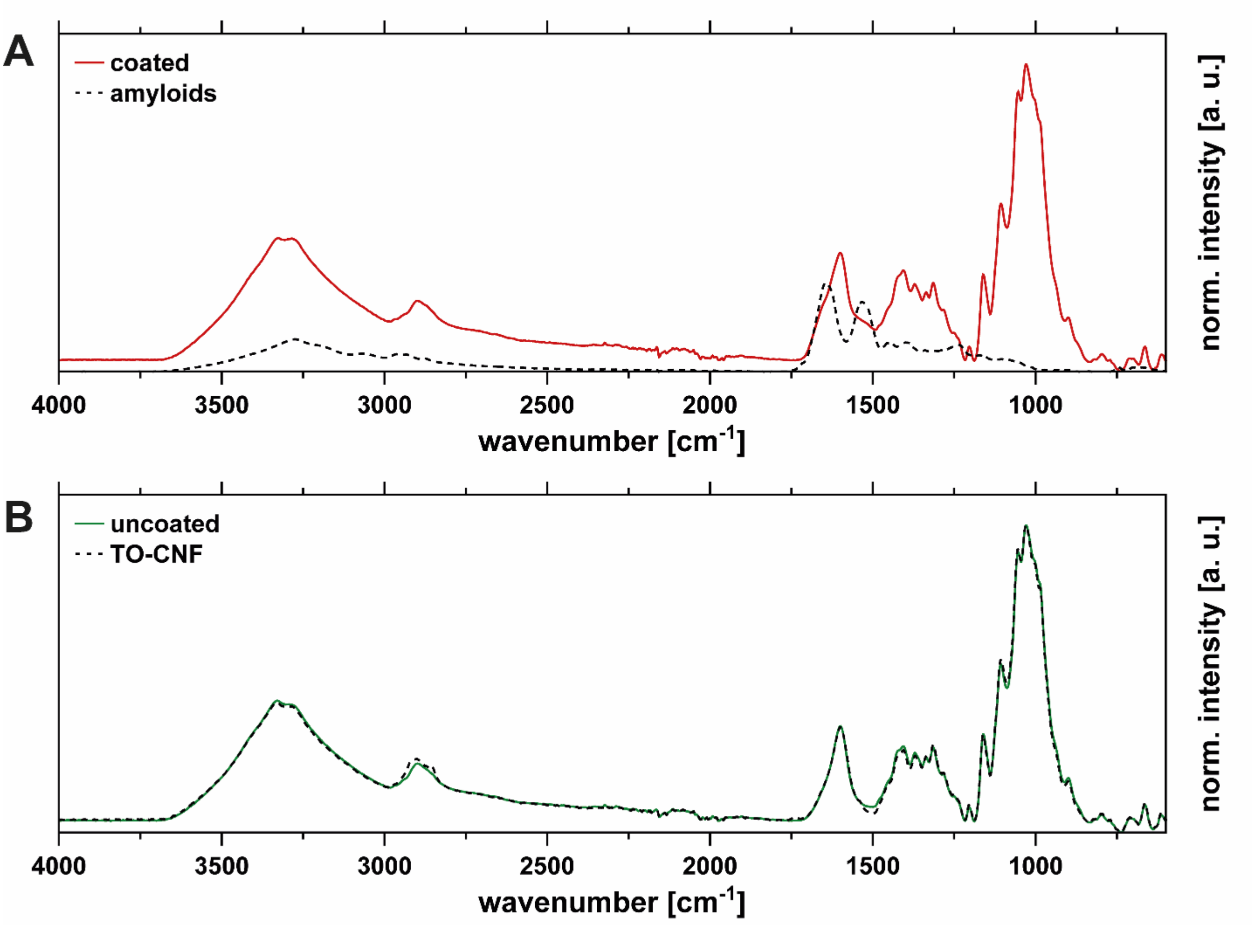

Figure S5: Full FTIR spectra of the side of the TO-CNF nanopaper coated with HEWL amyloid fibrils in red and reference dried HEWL amyloid fibril suspension in black (A). Full FTIR spectra of the uncoated side of the TO-CNF nanopaper in green and reference dried TO-CNF suspension in black (B).

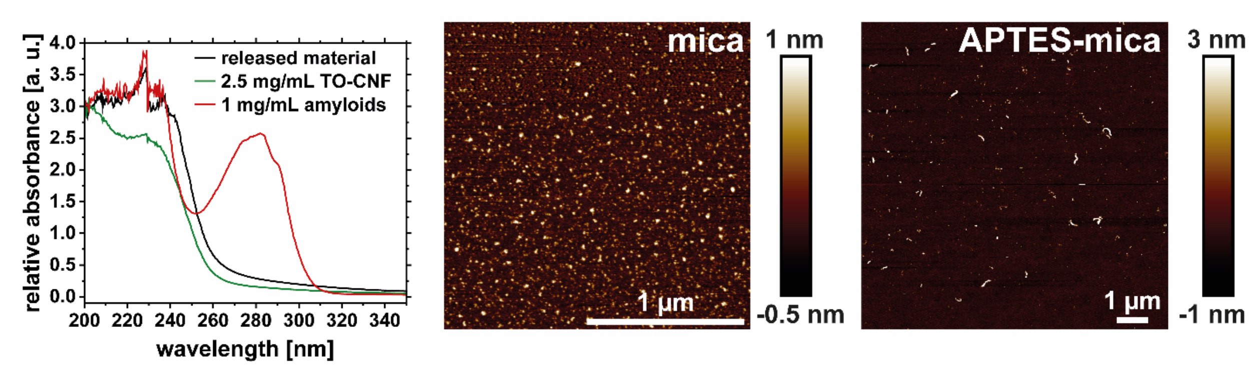

Figure S6: UV-vis spectrum of the material released from the TO-CNF nanopaper coated with HEWL amyloids (immersion of the film in MilliQ water for 48 h). For reference spectra of a 1 mg/mL amyloid fibril solution and a 2.5 mg/mL TO-CNF suspension are shown. The AFM images show the released material adsorbed onto freshly cleaved mica (negatively charged, adsorbing only positively charged peptides released from the coated nanopaper) and APTES-modified mica (positively charged, adsorbing negatively charged TO-CNFs released).

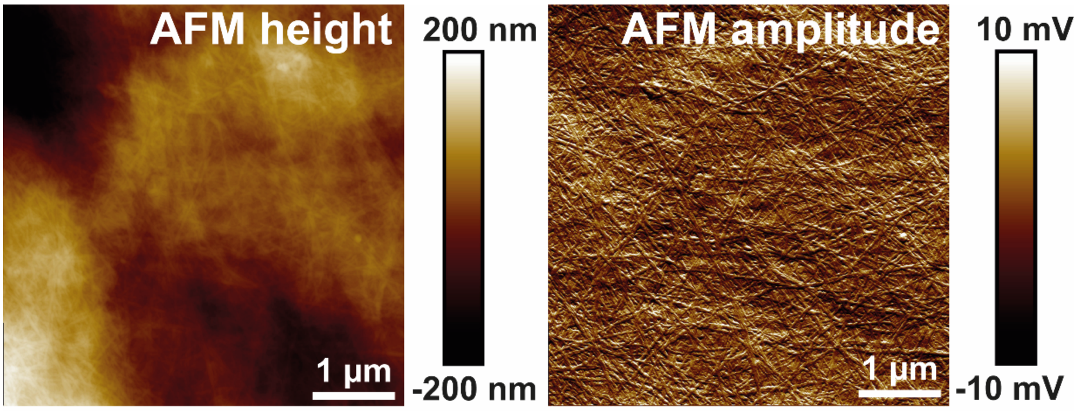

Figure S7: AFM height and amplitude images of a TO-CNF nanopaper coated with HEWL amyloids after immersion in MilliQ water and drying.
